# Evaluation of Alpha-Ketoglutarate Supplementation on the Improvement of Intestinal Antioxidant Capacity and Immune Response in Songpu Mirror Carp (*Cyprinus carpio*) After Infection With *Aeromonas hydrophila*

**DOI:** 10.3389/fimmu.2021.690234

**Published:** 2021-06-18

**Authors:** Di Wu, Ze Fan, Jinnan Li, Yuanyuan Zhang, Chang’an Wang, Qiyou Xu, Liansheng Wang

**Affiliations:** ^1^ Key Laboratory of Aquatic Animal Diseases and Immune Technology of Heilongjiang Province, Heilongjiang River Fisheries Research Institute, Chinese Academy of Fishery Sciences, Harbin, China; ^2^ School of Life Science, Huzhou University, Huzhou, China

**Keywords:** alpha-ketoglutarate, antioxidant capacity, immune response, Songpu mirror carp (*Cyprinus carpio*), *Aeromonas hydrophila*

## Abstract

As an intermediate substance of the tricarboxylic acid cycle and a precursor substance of glutamic acid synthesis, the effect of alpha-ketoglutarate on growth and protein synthesis has been extensively studied. However, its prevention and treatment of pathogenic bacteria and its mechanism have not yet been noticed. To evaluate the effects of alpha-ketoglutarate on intestinal antioxidant capacity and immune response of Songpu mirror carp, a total of 360 fish with an average initial weight of 6.54 ± 0.08 g were fed diets containing alpha-ketoglutarate with 1% for 8 weeks. At the end of the feeding trial, the fish were challenged with *Aeromonas hydrophila* for 2 weeks. The results indicated that alpha-ketoglutarate supplementation significantly increased the survival rate of carp after infection with *Aeromonas hydrophila* (*P* < 0.05), and the contents of immune digestion enzymes including lysozyme, alkaline phosphatase and the concentration of complement C4 were markedly enhanced after alpha-ketoglutarate supplementation (*P* < 0.05). Also, appropriate alpha-ketoglutarate increased the activities of total antioxidant capacity and catalase and prevented the up-regulation in the mRNA expression levels of pro-inflammatory cytokines including tumor necrosis factor-α, interleukin-1β, interleukin-6, and interleukin-8 (*P* < 0.05). Furthermore, the mRNA expression levels of toll-like receptor 4 (TLR4), and nuclear factor kappa-B (NF-κB) were strikingly increased after infection with *Aeromonas hydrophila* (*P* < 0.05), while the TLR4 was strikingly decreased with alpha-ketoglutarate supplementation (*P* < 0.05). Moreover, the mRNA expression levels of tight junctions including claudin-1, claudin-3, claudin-7, claudin-11 and myosin light chain kinases (MLCK) were upregulated after alpha-ketoglutarate supplementation (*P* < 0.05). In summary, the appropriate alpha-ketoglutarate supplementation could increase survival rate, strengthen the intestinal enzyme immunosuppressive activities, antioxidant capacities and alleviate the intestinal inflammation, thereby promoting the intestinal immune responses and barrier functions of Songpu mirror carp *via* activating TLR4/MyD88/NF-κB and MLCK signaling pathways after infection with *Aeromonas hydrophila*.

## Introduction

Extensive aquaculture practices subject fish to environmental stress, increasing the susceptibility to various pathogens (1). As a worldwide concern of human-animal-fish co-pathogenic bacteria, *Aeromonas hydrophila* (*A. hydrophila*, Ah) is able to cause septicemia in fish and acute diarrhea in children, leading to result in the huge pressure and losses of human health and aquaculture industry every year (2). Accordingly, antimicrobial agent or immunotherapeutic agent that can effectively prevent and cure pathogenic bacteria is one of great significance to the healthy growth of aquatic animals and even humans.

Alpha-ketoglutarate (AKG), as an intermediate in the tricarboxylic acid cycle of organisms and a precursor to glutamine, is the most important energy source of the gastrointestinal tract, especially as the repair stage of intestinal mucosa injury, which has the double effects of curing and preventing disease (3–5). Exogenous AKG has been reported to promote absorption of nutrients and even modulate intestinal amino acid metabolism and immunity (6, 7). In recent years, the effects of AKG on intestinal energy status and anti-oxidative capacity have been studied in ducks (8), rats (9), fruit flies (10) and piglets (11). However, a handful of studies have been reported the clinical benefits of AKG in improving immunity in malnutrition or inflammatory diseases to maintain intestinal structure and function under stress (12, 13). These observations indicated that AKG supplementation may has beneficial effects on the intestinal innate immune response of fish, and this possibility is worthy of study with respect to aquatic animals. As one of the main farmed aquaculture species in China, Songpu mirror carp (*Cyprinus carpio*), a kind of common carp which was selected based on the F4 generation breeding lines of Germany mirror carp, has great commercial value and is widely distributed worldwide (14). Our previous studies on Songpu mirror carp have shown that AKG had a beneficial effect on increasing the growth performance and protein efficiency (15, 16), while information regarding the effects of AKG supplementation on innate immune response in Songpu mirror carp is scarce, and little is known about the molecular mechanisms responsible for the action of AKG on the intestine of carp.

Several studies have shown that appropriate AKG levels can improve immunity in aquatic animals, and that supplementation of dietary AKG may activate the NF-kB, thereby upregulating the expression of pro-inflammatory cytokines and downregulating the expression of anti-inflammatory cytokines (17–19). However, the important thing that gets overlooked is that toll-like receptor 4 (TLR4), as one of the important receptors for inflammation recognition, activates the myeloid differentiation factor 88 (MyD88)-dependent pathway, which in turn activates nuclear factor kappa B (NF-κB), and ultimately leads to the release of inflammatory mediators and cytokines, thus playing an anti-inflammatory immunomodulatory role (20, 21). A growing number of studies have shown that the TLR4/MyD88/NF-κB signaling pathways play a key role in the pathogenesis and treatment of a variety of diseases (22–24). The innate immune response induces an inflammatory response, which is primarily mediated by cytokines, and the crucial pro-inflammatory cytokines, such as tumor necrosis factor α (TNF-α) and interleukin 8 (IL-8), possess the capabilities of increasing the synthesis of small inflammatory mediators and initiating inflammatory processes. Conversely, the anti-inflammatory cytokines interleukin 10 (IL-10) and transforming growth factor-β (TGF-β) are produced to inhibit the excessive activation of the inflammatory response and initiate tissue recovery processes (25). Furthermore, intestinal immune function in fish is also tightly associated with intestinal barrier function (26). Myosin light chain kinase (MLCK) plays an important role in tight junction permeability and immune health by regulating the protein expressions of claudins, occludins and zonula occludens (ZOs), leading to the increase of intercellular permeability (27). Improving the mechanical barrier and immune barrier function of the intestinal tract of fish is a necessary condition for ensuring intestinal health (28, 29). Previous studies mostly focused on the effect of the NF-kB signaling pathway on inflammatory factors, but ignored the regulatory effect of the upstream molecule TLR4 on NF-kB. Therefore, we suspected that whether the effect of AKG on immunity through the both TLR4/MyD88/NF-kB and MLCK signaling pathways. Moreover, the intestinal mucosal immune function primarily depends on intestine-associated lymphoid tissue that consists of variably sized immune cells such as immune enzymes and complements (30, 31). The immune cells are capable of secreting humoral components, which play crucial roles in the intestinal innate immune response of fish (32). Similarly, to other vertebrates, fish attempt to minimize oxidative damage *via* antioxidant defense system (33). Hence, we thus focused on intestinal immune-related enzymes, intestinal antioxidant capacities, and physical barrier functions, regulated *via* the TLR4/MyD88/NF-kB and MLCK signaling pathway by using Songpu mirror carp with AKG supplementation after infection with *A. hydrophila*. At the same time, the positive effect of AKG as antibacterial agent or immunotherapeutic agent was further evaluated.

## Materials and Methods

### Experimental Diet and Procedures

The formulation and approximate composition of the experimental diets are specified in the formulation of basal diet as shown in **Table 1**. Fish meal and soya bean meal were used as dietary protein sources. Fish oil, soya bean oil and phospholipid were used as dietary lipid sources. According to the principles of equal nitrogen and equal energy, 1% AKG (Sigma-Aldrich, Shanghai, China) was used to replace glucose in the basic feed. All ingredients were crushed and pulverized with an 80 mesh sieve. After weighing the ingredients according to the formula, all of the ingredients were homogenized in a mixer. Distilled water was added to achieve a proper pelleting consistency, and the mixture was further homogenized and formed into 1-mm pellets using a pellet machine. After being prepared completely, the diets were stored at -20°C until using.

**Table 1:** Composition and nutrients content of diets.

Ingredients	Contents (g/kg)	
	Control^e^	AKG^f^
Fish meal	80.00	80.00
Soya bean meal	560.00	560.00
Wheat middling	230.00	230.00
Fish oil	10.00	10.00
Soya bean oil	33.00	33.00
Phospholipid	10.00	10.00
Vitamin premix^a^	3.00	3.00
Mineral premix^b^	2.00	2.00
AKG^c^	0.00	1.00
Choline chloride	5.00	5.00
NaH_2_PO_4_	20.00	20.00
Limestone	10.00	10.00
Methionine	3.00	3.00
Threonine	3.00	3.00
Cr_2_O_3_	1.00	1.00
CMCC	15.00	15.00
Glucose	15.00	14.00
**Nutrient levels**		
Crude protein^d^	383.80	383.80
Ether extract^d^	62.30	62.30
Total phosphorus	8.50	8.50
Lysine	22.00	22.00
Methionine	8.00	8.00
Threonine	16.70	16.70

^a^The vitamin premix provided the following per kg of the diet: VA 8,000 IU, VC 500 mg, VD_3_ 3,000 IU, VE 60 mg, VK_3_ 5 mg, VB_2_ 30 mg, VB_6_ 15 mg, VB_12_ 0.5 mg, choline chloride 5,000 mg, nicotinic acid 175 mg, D-biotin 2.5 mg, inositol 1,000 mg, folic acid 5 mg, pantothenic acid 50 mg.

^b^The mineral premix provided the following per kg of the diet: Zn 25 mg, Cu 3 mg, Fe 25 mg, Mn 15 mg, I 0.6 mg, Co 0.1 mg, Se 0.4 mg.

^c^AKG (alpha-ketoglutarate) with a purity of 98%.

^d^Crude protein and ether extract were measured values, while the others were calculated values.

^e^Control, basal diet.

^f^AKG, basal diet supplemented with 1% AKG.

### Feeding Trial

Songpu mirror carp (*Cyprinus carpio*) were obtained from Heilongjiang River Fisheries Research Institute in Harbin, Heilongjiang, China. Before starting the experiment, the fish were acclimated to experimental conditions for 2 weeks and fed with a basal diet three times daily. After acclimation, 360 carp with an average initial weight of 6.54 ± 0.08 g were randomly assigned to 12 experimental tanks (1.2 m length × 1.2 m width × 0.8 m height), resulting in 30 carp per tank. Meanwhile, the carp were divided into two groups: (1) control groups (CK group, carp fed with basal diet); (2) AKG groups (AKG group, carp fed with basal diet supplemented with 1% AKG). Each group has 6 experimental tanks of replicates at the period of feeding trial. The experiment at the period of feeding trial was carried out in an indoor aquarium with a controlled water circulation system. All groups of fish were fed with their respective diets at the same fixed rate approaching satiation means that the daily feeding amount is about 5% of body weight, and divided into equal portions three times (8:30, 12:30 and 16:30) for 8 weeks. The fish were counted and weighed every 2 weeks from the onset of the experiment trial to check the body weight and to adjust the amount of feed. During the experimental period, dissolved oxygen exceeded 6.0 mg/L, and the water temperature was set as 23.0 ± 0.5°C, while the pH value was at 7.4 ± 0.3. The NO_2_-N and NH_4_^+^-N concentrations were not in excess of 0.02 and 0.5 mg/L, respectively. At the same time, one third of the water was renewed per day to ensure water quality, and the feeding trials were conducted under the natural light and dark cycle according to the method of Wang et al. (34).

At the end of an 8-week feeding trial, 3 carp from each tank (a total of 18 carp from each group) were selected and their body weight and length were measured, while their feed intake was also recorded. The collected data were used to calculate the following parameters:

$$\mathrm{Weight growth rate}(\text{WGR},\;\%)\;=\;\frac{W_{t}-W_{0}}{W_{0}}\times 100\%$$

$$\text{Feed conversion ratio}\;(\text{FCR})\;=\;\frac{F}{W_{t}-W_{0}}$$

where *W_t_* and *W*_0_ are the final and initial body weights (g), respectively; *F* is the feed consumed (g).

### Challenge Trial

After feeding trial, fish were deprived of food for 24 h before infection with *A. hydrophila*. The infection assay was performed as described by Chen et al. (35) with slight modification. *A. hydrophila* strains used during the challenged experiment were preserved in our laboratory. The strains were cultured with broth medium for 24 h in an incubator at 37°C, and were rejuvenated twice. Then, the bacteria in the logarithmic growth phase was selected, centrifuged at 2500 g/min for 1 min, and the precipitated bacteria was collected and washed with sterilized normal saline. After collecting the bacteria, the dosage was determined by the semilethal concentration 50 (LC50) value for carp in a pre-experiment.

During the challenge trial, according to the injection of *A. hydrophila* or sterilized normal saline (NS), carp in the control groups were divided into two subgroups: (1) control group with non-challenged (CK + NS group, carp fed with basal diet and receiving an intraperitoneal administration of sterile saline); (2) *A. hydrophila*-challenged group (CK + Ah group, carp fed with basal diet and receiving an intraperitoneal administration of *A. hydrophila*). Similarly, carp in the AKG groups were divided into two subgroups: (3) AKG group with non-challenged (AKG + NS group, carp fed the basal diet supplemented with 1% AKG and receiving an intraperitoneal administration of sterile saline); (4) AKG group with *A. hydrophila-*challenged (AKG + Ah) group, carp fed the basal diet supplemented with 1% AKG and receiving an intraperitoneal administration of *A. hydrophila*). Each subgroup has 3 experimental tanks of replicates at the period of challenge trial. Carp in the CK + Ah and AKG + Ah groups were intraperitoneally injected with 0.2 mL of 2.0 ×10^7^ colony-forming units (CFU)/mL *A. hydrophila* for each individual, whereas carp in the CK + NS and AKG + NS groups were intraperitoneally injected with equal volumes of sterilized normal saline. Then, the fish were returned to each experimental tank for 2 weeks. During the infection, carp was fed with basal diet, and the water is constantly aerated, while the water does not circulate. Replace one-third of the water in the bucket every three days and inject new water to ensure the quality of the water. The other experimental conditions were the same as the feeding trial during the challenge trial. The symptoms of infected fish were observed, and the dead fish were caught out in time, and the mantissa of death was recorded. The collected data were used to calculate the following parameter:

$$\text{Survival rate}(\text{SR},\;\%)\;=\;\frac{N_{t}}{N_{0}}\times 100\%$$

where *N_t_* and *N*_0_ are the final and initial numbers of carp, respectively.

### Sample Collection

At the termination of challenge trial, 9 carp per tank (27 carp per subgroups) randomly selected from each treatment group were anaesthetized with tricane methane sulphonate MS-222 (75 mg/L) as described by Wang et al. (34). The intestine of carp sample was quickly removed and the sampling site, the mid intestine (MI), was selected between the first and last turns of the whole intestine. The MI of 6 carp per tank were preserved and stored at -40°C for biochemical analysis. The rest of 3 carp per tank were frozen in liquid nitrogen, and then stored at -80°C for gene transcription analysis.

### Biochemical Analysis

Intestinal sample was homogenized in 10 volume (w/v) of ice-cold saline and centrifuged at 6,000 g for 20 min at 4°C. Subsequently, the supernatants were used for biochemical analysis. The content of lysozyme (LZM) was measured using turbidimetry. The determination principles of acid phosphatase (ACP) and alkaline phosphatase (ALP) are that the free phenol produced after the decomposition of phenyl disodium phosphate by ACP and ALP can be oxidized by potassium ferricyanide to form red quinone derivatives with 4-aminoantipyrazine in alkaline solution. The activity of total superoxide dismutase (T-SOD) was measured following the hydroxylamine method. The concentration of total antioxidant capacity (T-AOC) was calculated according to ABTS method. The activity of glutathione peroxidases (GSH-Px) was assayed in a coupled enzyme system where NADPH is consumed by glutathione reductase to convert the formed GSSG to its reduced form (GSH). The activity of catalase (CAT) was detected by ammonium molybdenum acid method. The lipids peroxidation was analyzed in terms of malondialdehyde (MDA) equivalents using the thiobarbituric acid (TBA) reaction. The concentrations of complements C3 and C4 were assayed by using the immunoturbidimetry kits. Briefly, complements C3 and C4 were mixed with the antibody afforded by the kits and then an antigen-antibody complex was produced. The optical density (OD) value was measured at 340 nm. Compared with the values of the standards from the kits, the C3 and C4 contents were calculated in mg/g protein. The concentration of immunoglobulin M (IgM) was determined according to the instruction of the kit. All of the above biochemical analysis assays were performed using commercially available kits following the manufacturer’s protocols, which purchased from Nanjing Jiancheng Bioengineering Institute, Nanjing, China.

### Real-Time Polymerase Chain Reaction (RT-PCR) Analysis

Total RNA was extracted using the total RNA isolation system kit (Takara, Dalian, China) according to the manufacturer’s recommended protocol. The concentration of RNA was quantified with a spectrophotometer (NanoDrop 2000, Thermo Fisher, Germany). The RNA quality was determined by analyzing the RNA integrity through agarose gel electrophoresis and by confirming that the A260 nm/A280 nm absorbance ratio was between 1.8 and 2.0. Subsequently, RNA was reverse transcribed into cDNA using the PrimeScript™ RT reagent kit (Takara, Dalian, China) following the manufacturer’s instructions. The obtained cDNA templates were then stored at -80°C for later use. Quantitative real-time PCR (qPCR) was carried out on the a LightCycler^®^ 480 thermol cycler (Roche, Germany) in a total volume of 10 μL with the LightCycler^®^ 480 SYBR Green I Master (Roche, Germany) following the manufacturer’s protocol. All amplification reactions were run in triplicate. Before the qPCR amplification, the specificity and efficiency of the primers for the β-actin and target genes were detected by constructing a standard curve using serial dilution of cDNA, and the standard equation and correlation coefficient for each primer pair were determined. The specific primers of the β-actin gene and target genes are listed in **Table 2**. The relative mRNA expression of target genes were normalized to the β-actin mRNA, and determined using the 2^−ΔΔCT^ method.

**Table 2:** Real-time PCR primer sequences.

Target gene	Primer sequence Forward (5’→3’)	Primer sequence Reverse (5’→3’)	Accession number
TLR4^a^	TGTCGCTTTGAGTTTGAAT	TCCAGAATGATGATGATGATC	NW_017540541.1
MyD88^b^	AAGAGGATGGTGGTAGTCA	GAGTGCGAACTTGGTCTG	LN590716.1
NF-κB^c^	TATTCAGTGCGTGAAGAAG	TATTAAAGGGGTTGTTCTGT	LN590678.1
TNF-α^d^	AAGTCTCAGAACAATCAGGAA	TGCCTTGGAAGTGACATT	AJ311800
IL-1β^e^	AACTTCACACTTGAGGAT	GACAGAACAATAACAACAAC	KC008576
IL-6^f^	GACCAGCAGGTACGTCTCAACAC	TCCTTCATACGCCGTCATGTTCAC	LN590906.1
IL-8^g^	AAACTGAGAGTCGACGCATTG	TTTTCAATGACCTTCTTAACCCAG	EU011243.1
IL-10^h^	GCCAGCATAAAGAACTCG	CCAAATACTGCTCGATGT	JX524550.1
TGF-β^i^	GGGACATCATCGCCATCT	TGACATTCTCGGCAGGGT	U66874.1
claudin-1	GACAACATCRTSACVGCHCAG	CMYTYCCRAACTCATACCT	LN598389.1
claudin-3	GCACCAACTGTATCGAGGATG	GGTTGTAGAAGTCCCGAATGG	LN590711.1
claudin-7	CTTCTATAACCCCTTCACACCAG	ACATGCCTCCACCCATTATG	LN591006.1
claudin-11	TCGGAAGTGAACCAGAAAGC	GAAGCCAAAGGACATCAAGC	LN590700.1
occludin	ATCGGTTCAGTACAATCAGG	GACAATGAAGCCCATAACAA	LN590695.1
ZO-1^j^	GCCTGCCTACACTCAACCACAAC	CTGCTTCGGCTGGAGGAGGAG	LN590708.1
MLCK^k^	CGATGGTGGCAGTGCTGTGAC	GACTCTTGGCTCGGTTCGCTAAC	LN590717.1
β-actin	GATCGGCAATGAGCGTTTCC	ACGGTGTTGGCATACAGGTC	M24113.1

^a^TLR4, toll-like receptor 4.

^b^MyD88, myeloid differentiation factor 88.

^c^NF-κB, nuclear factor kappa-B.

^d^TNF-α, tumor necrosis factor-α.

^e^IL-1β, interleukin-1β.

^f^IL-6, interleukin-6.

^g^IL-8, interleukin-8.

^h^IL-10, interleukin-10.

^i^TGF-β, transforming growth factor-β.

^j^ZO-1, zonula occludens-1.

^k^MLCK, myosin light chain kinases.

### Statistical Analysis

Results were calculated using the statistical software SPSS 22.0 (SPSS Inc., Chicago, IL, USA). All data were subjected to two-way analysis of variance (ANOVA) to determine whether significant differences occurred with respect to AKG and *A. hydrophila*, or any interaction between these two factors. If a significant difference was identified, the data were reanalyzed by one-way analysis and Tukey’s *post hoc* test. Results are presented as mean values and the standard error of the mean (SEM). Differences were considered significant if *P* < 0.05.

## Results

### The Survival Rate of Songpu Mirror Carp After Infection With *A. hydrophila*

**Figure 1:**
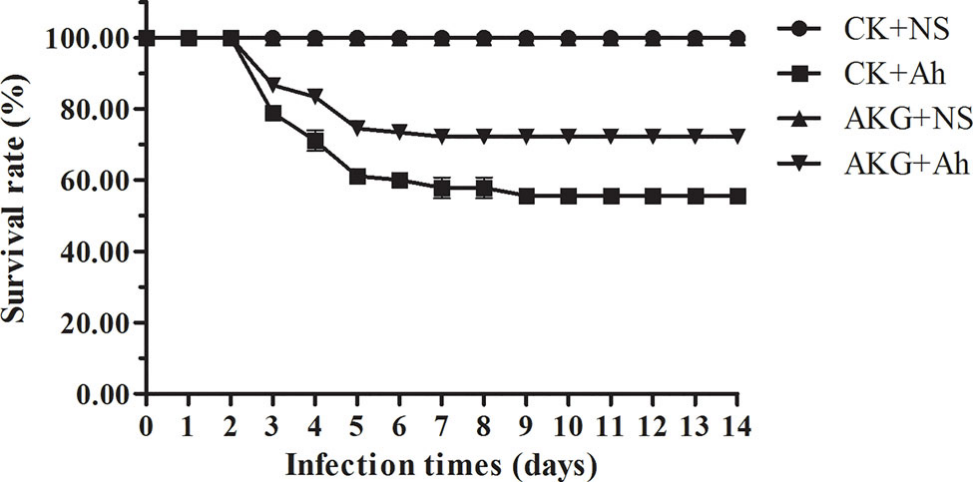
Effects of AKG on survival rate of *A. hydrophila* infected-Songpu mirror carp. Survival data was registered for uninfected Songpu mirror carp (*Cyprinus carpio*) fed with or without AKG supplementation as well as *A. hydrophila*-challenged Songpu mirror carp fed with or without AKG supplementation. The number of carp was 30 per subgroup.

After injection with *A. hydrophila*, the first mortality was recorded after 12 h. As shown in **Figure 1**, *A. hydrophila*-challenged carp in showed a survival of 55.56 ± 1.92% in the CK +Ah group at the end of challenge trial. The dietary AKG supplementation causes a survival of 72.22 ± 1.92% in the AKG +Ah group at the end of challenge trial. Statistical analysis revealed a significant increase in the survival of *A. hydrophila*-challenged carp in the AKG +Ah group compared with carp in the CK +Ah group (*P* < 0.05). In addition, the carp injection with *A. hydrophila* had bleeding symptoms at the injection site and the base of the fin began on the second day, and the *A. hydrophila-*challenged carp sought for more oxygen on the water surface, exhibiting slow and unbalanced moving, some even roll over when compared to uninfected ones.

### Growth Performance of Songpu Mirror Carp With AKG Supplementation

The effects of AKG supplementation on the growth and feed utilization of Songpu mirror carp at the end of feeding trial are presented in **Table 3**. The final body weight (FBW) and weight growth rate (WGR) in the AKG groups were significant higher than those in the control groups (*P* < 0.05). And AKG supplementation significantly reduced the feed conversion ratio (FCR) of carp in the AKG groups compared with the control groups (*P* < 0.05).

**Table 3:** The effects of AKG on the growth and feed utilization of Songpu mirror carp (*Cyprinus carpio*).

Groups	IBW^a^ (g)	FBW^b^ (g)	WGR^c^ (%)	FCR^d^
Control^e^	6.53 ± 0.06	51.52 ± 0.89^b^	689.53 ± 12.22^b^	1.36 ± 0.01^a^
AKG^f^	6.55 ± 0.04	59.34 ± 1.17^a^	806.69 ± 16.37^a^	1.29 ± 0.02^b^

All of the values are expressed as the means ± SEM, and different superscript letters in the same column are significantly different (P < 0.05).

^a^IBW, initial body weight (g).

^b^FBW, final body weight (g).

^c^WGR, weight gain rate (%).

^d^FCR, feed conversion ratio.

^e^Control, the control groups during the feeding trial.

^f^AKG, the AKG groups during the feeding trial.

### Intestinal Immune-Related Enzymes of Songpu Mirror Carp After Infection With *A. hydrophila*

**Figure 2:**
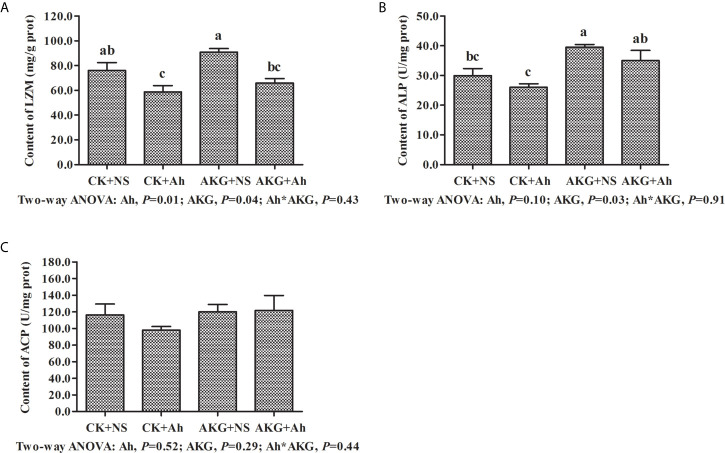
Effects of AKG supplementation and *A hydrophila* infection on immune-related enzymes in intestine of Songpu mirror carp (*Cyprinus carpio*). **(A)** LZM, lysozyme; **(B)** ALP, alkaline phosphatase; **(C)** ACP, acid phosphatase. All of the values are expressed as the means ± SEM. Values with different letters are significantly different (*P* < 0.05).

The effects of AKG supplementation and *A. hydrophila* infection on immune-related enzymes in the intestine of Songpu mirror carp at the end of challenge trial are presented in **Figure 2**. The content of LZM in the CK + Ah group was significantly decreased compared with that in the CK + NS group (*P* < 0.05). No significant differences were found in content of ALP between the CK + NS group and AKG + Ah group (*P* > 0.05), while the content of ALP in the AKG + Ah group was significantly increased compared with that in the CK + Ah group (*P* < 0.05). AKG supplementation or *A. hydrophila* infection exerted no significant effects on the content of ACP, even though it exhibited a tendency to increase (*P* > 0.05). The contents of LZM and ALP were significantly elevated upon AKG supplementation into the feed (*P* < 0.05), and there was no obvious interaction between AKG supplementation and *A. hydrophila* infection on LZM, ALP and ACP in the intestine of carp (*P* > 0.05).

### Intestinal Immune-Related Complements of Songpu Mirror Carp After Infection With *A. hydrophila*

**Figure 3:**
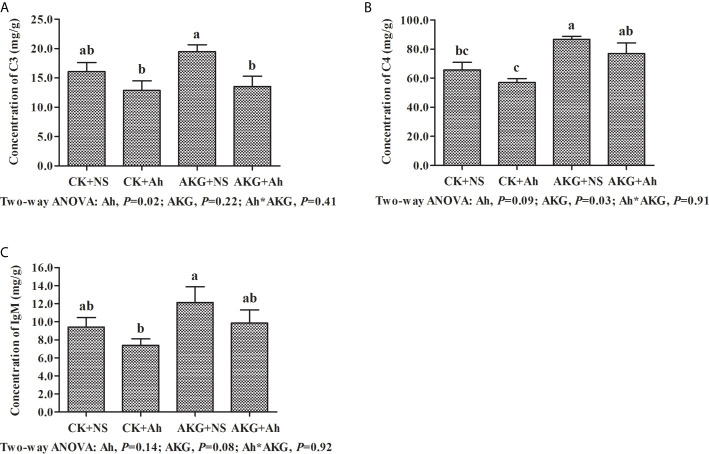
Effects of AKG supplementation and *A hydrophila* infection on immune-related parameters complements in intestine of Songpu mirror carp (*Cyprinus carpio*). **(A)** complement C3; **(B)** complement C4; **(C)** IgM, immunoglobulin M. All of the values are expressed as the means ± SEM. Values with different letters are significantly different (*P* < 0.05).

The effects of AKG supplementation and *A. hydrophila* infection on the concentrations of complements C3 and C4 and the concentration of IgM in the intestine of Songpu mirror carp at the end of challenge trial are displayed in **Figure 3**. Compared with the AKG+Ah group, the concentration of complement C3 in the AKG + NS group was significantly increased (*P* < 0.05), and the concentration of complement C4 in the AKG + NS group was significantly higher than that in the CK + NS group and CK + Ah group, respectively (*P* < 0.05). The concentration of complement C4 in the AKG + Ah group was significantly increased compared with the CK+Ah group (*P* < 0.05). The concentration of IgM in the AKG + NS group was higher than that in the CK + Ah group (*P* < 0.05). There was no obvious interaction between AKG supplementation and *A. hydrophila* infection on the concentrations of complements C3 and C4 and the concentration of IgM in the intestine of carp (*P* > 0.05).

### Antioxidant-Related Parameters of Songpu Mirror Carp After Infection With *A. hydrophila*

The effects of AKG supplementation and *A. hydrophila* infection on intestinal antioxidant capacities of Songpu mirror carp at the end of challenge trial are presented in **Table 4**. The activity of T-AOC in the AKG + NS group was significantly higher compared with the CK + NS, CK + Ah and AKG + Ah groups (*P* < 0.05), and the AKG + NS group could even significantly decrease the content of MDA compared with the CK + Ah and AKG + Ah group (*P* < 0.05). The results indicated that the activities of SOD, CAT and T-AOC were significantly decreased after infection with *A. hydrophila* (*P* < 0.05), and the supplementation of AKG generated a remarkable increase of the activities of CAT and T-AOC (*P* < 0.05). In addition, the content of MDA in the intestine of Songpu mirror carp significantly increased after infection with *A. hydrophila* (*P* < 0.05). There was no significant interaction between AKG supplementation and *A. hydrophila* infection on the activities of SOD, CAT, GSH-Px and MDA (*P* > 0.05), while AKG supplementation and *A. hydrophila* infection exhibited a significant interaction on the activity of T-AOC in the intestine of carp (*P* < 0.05).

**Table 4:** Effects of AKG supplementation and *A. hydrophila* infection on antioxidant-related parameters in intestine of Songpu mirror carp (*Cyprinus carpio*).

Groups	SOD^a^ (U/mg protein)	CAT^b^ (U/mg protein)	GSH-Px^c^ (U/mg protein)	T-AOC^d^ (U/mg protein)	MDA^e^ (nmol/g tissues)
CK+NS	113.34 ± 16.94^ab^	38.70 ± 4.87^ab^	79.74 ± 13.61	0.91 ± 0.12^b^	4.27 ± 0.64^c^
CK+Ah	95.55 ± 8.03^b^	31.18 ± 2.95^b^	76.81 ± 6.95	0.69 ± 0.05^b^	7.50 ± 0.87^a^
AKG+NS	125.23 ± 16.25^a^	44.74 ± 6.28^a^	75.85 ± 9.65	1.26 ± 0.21^a^	3.47 ± 0.44^c^
AKG+Ah	103.43 ± 11.48^ab^	39.68 ± 2.71^ab^	73.33 ± 11.75	0.73 ± 0.09^b^	5.73 ± 0.28^b^
*P*-value of two-way ANOVA					
Ah	0.04	0.04	0.67	0.01	0.00
AKG	0.25	0.02	0.57	0.03	0.01
Ah*AKG	0.81	0.65	0.91	0.08	0.20

All of the values are expressed as the means ± SEM, and different superscript letters in the same column are significantly different (P < 0.05).

^a^SOD, superoxide dismutase.

^b^CAT, catalase.

^c^GSH-Px: glutathione peroxidase.

^d^T-AOC: total antioxidant capacity.

^e^MDA, malondialdehyde.

### Immune Response-Related Signaling Pathways of Songpu Mirror Carp After Infection With *A. hydrophila*

**Figure 4:**
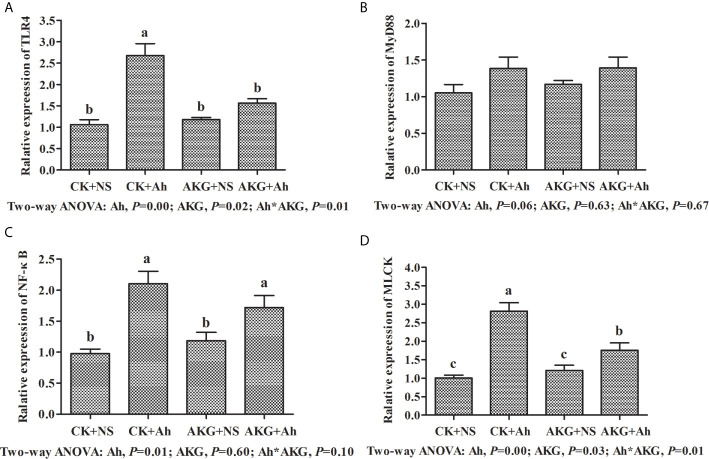
Effects of AKG supplementation and *A hydrophila* infection on mRNA expression levels of immune response-related signaling pathways in intestine of Songpu mirror carp (*Cyprinus carpio*). **(A)** TLR4, toll-like receptor 4; **(B)** MyD88, myeloid differentiation factor 88; **(C)** NF-κB, nuclear factor kappa-B; **(D)** MLCK, myosin light chain kinases. All of the values are expressed as the means ± SEM. Values with different letters are significantly different (*P* < 0.05).

As shown in **Figure 4**, the gene expression levels of TLR4 and NF-κB in the CK + Ah group were significantly upregulated compared with those in the CK + NS group (*P* < 0.05), and the AKG + Ah group was associated with a significant downward trend of the gene expression levels of TLR4 compared with the CK + Ah group (*P* < 0.05). In addition, the gene expression level of TLR4 in the AKG + Ah group was significantly downregulated compared with the CK + Ah group (*P* < 0.05). Moreover, the mRNA expression level of MLCK in the CK + Ah group was significantly upregulated compared with the CK + NS, AKG + NS and AKG + Ah groups (*P* < 0.05), and the mRNA expression level of MLCK in the AKG + Ah group was significantly higher than that in the CK + NS and AKG + NS group (*P* < 0.05). The gene expression levels of TLR4 and NF-κB were significantly upregulated after infection with *A. hydrophila* (*P* < 0.05), and AKG supplementation was associated with a significant downward trend of the gene expression levels of TLR4 (*P* < 0.05). A significant interaction between AKG supplementation and *A. hydrophila* infection on the gene expression levels of TLR4 and MLCK were showed in the intestine of carp (*P* < 0.05), while there was no interaction on the gene expression levels of MyD88 in the intestine of carp (*P* > 0.05).

### Relative mRNA Expression Levels of TLR4/MyD88/NF-κB Signaling Pathways of Songpu Mirror Carp

**Figure 5:**
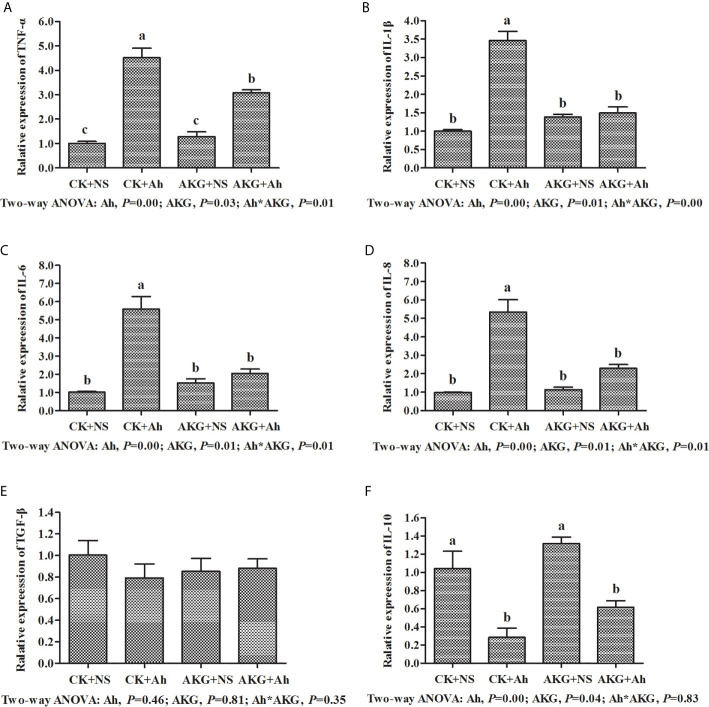
Effects of AKG supplementation and *A hydrophila* infection on relative mRNA expression levels of immune-related TLR4/MyD88/NF-κB signaling pathways of Songpu mirror carp (*Cyprinus carpio*). **(A)** TNF-α, tumor necrosis factor-α; **(B)** IL-1β, interleukin-1β; **(C)** IL-6, interleukin-6; **(D)** IL-8, interleukin-8; **(E)** TGF-β, transforming growth factor-β; **(F)** IL-10, interleukin-10. All of the values are expressed as the means ± SEM. Values with different letters are significantly different (*P* < 0.05).

To investigate the effects of AKG supplementation and *A. hydrophila* infection on the intestinal inflammatory response in carp, the expression levels of TNF-α, IL-1, IL-6, IL-8, IL-10 and TGF-β genes in the intestines were determined at the end of challenge trial. The mRNA levels of inflammatory cytokines and related signaling molecules mRNA levels in the intestine of Songpu mirror carp are presented in **Figure 5**. The gene expression levels of TNF-α, IL-1β, IL-6 and IL-8 in the CK + Ah group were significantly higher than those of the CK + NS group (*P* < 0.05). In addition, the mRNA expression levels of above pro-inflammatory cytokines with AKG supplementation groups exhibited the opposite results after infection with *A. hydrophila*. The pro-inflammatory expression levels of TNF-α, IL-1β, IL-6 and IL-8 in the in the AKG + Ah group were significantly downregulated compared with those in the CK + Ah group (*P* < 0.05). For anti-inflammatory factors, the mRNA expression level of IL-10 reached the minimum value after infection with *A. hydrophila*. In contrast, the mRNA expression level of IL-10 was upregulated with AKG supplementation and reached the maximum value in the AKG + NS group. The mRNA expression level of TGF-β in the intestine of carp exhibited no significant differences with respected to AKG supplementation or infection with *A. hydrophila* (*P* > 0.05). A significant interaction between AKG supplementation and *A. hydrophila* infection on the gene expression levels of TNF-α, IL-1β, IL-6 and IL-8 were showed in the intestine of carp (*P* < 0.05).

### Relative mRNA Expression Levels of MLCK Signaling Pathway of Songpu Mirror Carp

**Figure 6:**
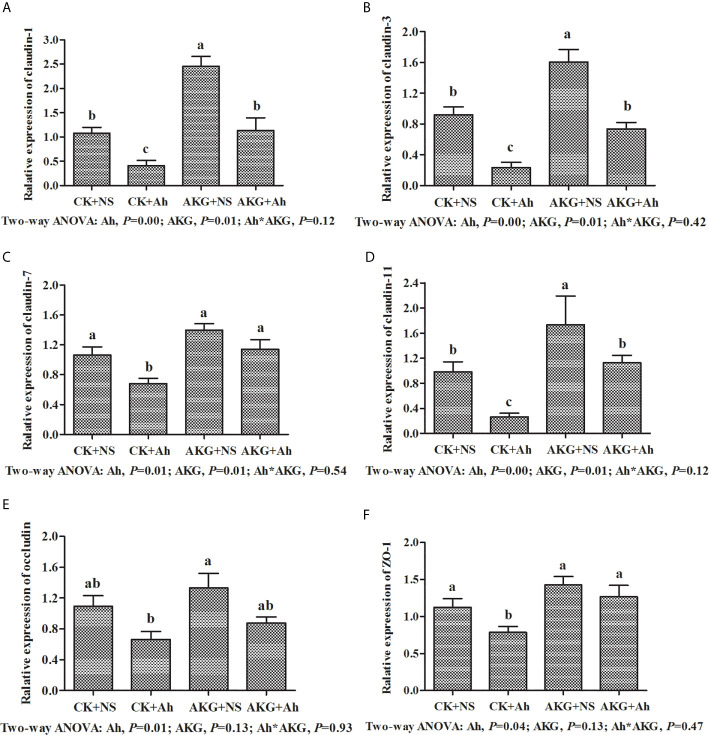
Effects of AKG supplementation and *A hydrophila* infection on relative mRNA expression levels of immune-related MLCK signaling pathway of Songpu mirror carp (*Cyprinus carpio*). **(A)** claudin-1; **(B)** claudin-3; **(C)** claudin-7; **(D)** claudin-7; **(E)** occludin; **(F)** ZO-1, zonula occludens-1. All of the values are expressed as the means ± SEM. Values with different letters are significantly different (*P* < 0.05).

To investigate the effects of AKG supplementation and *A. hydrophila* infection on the barrier function in Songpu mirror carp, the expressions of claudin-1, claudin-3, claudin-7, claudin-11, occludin and ZO-1 in the intestine of Songpu mirror carp were determined at the end of challenge trial. As shown in **Figure 6**, the mRNA levels of claudin-1, claudin-3, claudin-7, claudin-11, occludin and ZO-1 were all downregulated in the case of infection with *A. hydrophila* (*P* < 0.05). In contrast, compared with infection groups, AKG supplementation could significantly upregulate the gene expression levels of claudin-1 and claudin-3, and as well as the mRNA level of claudin-7 and claudin-11 (*P* < 0.05). However, AKG supplementation exerted no effects on the mRNA expression levels of occludin and ZO-1 (*P* > 0.05). There was no significant interaction between AKG supplementation and *A. hydrophila* infection on the mRNA expression levels of claudin-1, claudin-3, claudin-7, claudin-11, occludin and ZO-1 of Songpu mirror carp (*P* > 0.05).

## Discussion

With the intensification and scale-up of the aquaculture industry, the problem of destruction of aquatic water and ecological environment has become increasingly prominent, and aquatic animal diseases have also frequently erupted (36, 37). *A. hydrophila*, which is an emergent bacterial pathogen that is commonly encountered in freshwater, was selected as the inflammatory infection model of Songpu mirror carp in this study. Subsequently, we systematically and comprehensively evaluated the molecular mechanisms of AKG which has been shown to have intestinal immunomodulatory effects as an immune drug through growth performance, non-specific immune enzymes, antioxidant system and immune barrier related signal pathways for the intestinal inflammation in the *A. hydrophila*-challenged carp.

First of all, we observed that *A. hydrophila-*challenged groups reduced concentrations of immune-related enzymes, while altering the concentrations of complements in carp. This is in keeping with our new findings that inflammatory cytokines of TNF-α, IL-1β, IL-6 and IL-8 were increased, as well as claudins and ZO-1 were reduced in response to *A. hydrophila* infection. In addition, *A. hydrophila* decreased the mirror carp survival during the weeks in this study. Moreover, the main external symptoms observed in *A. hydrophila*-challenged carp were ulceration and hemorrhagic focus that became disseminated through the fin base, mouth and gill slit. Moreover, it was noticed that *A. hydrophila*-challenged carp sought for more oxygen on the water surface, exhibiting abnormal swimming behavior and loss of balance. Thus, the *A. hydrophila*-challenged carp provide a useful animal model to develop nutritional interventions for improving intestinal status combined with the clinical symptoms that we observed after the injection. Accordingly, previous studies reported that *A. hydrophila* induces cumulative mortality (38) and declined survival rate (39) in different fish species. Encouragingly, AKG supplementation improved the reduced survival rate of the carp caused by *A. hydrophila* infection. Previous study has shown that AKG could reduce the mortality of *Eriocheir sinensis* caused by *Spiroplasma eriocheiris* infection (40). And the protective effects of dietary AKG to resist sodium nitroprusside and hydrogen peroxide toxicity have been proved on fruit flies (41). Previous studies were consistent with our results, that is, AKG has the ability to protect animals from exogenous pathogens.

Dietary AKG supplementation increased FBW and WGR and decreased FCR of Songpu mirror carp significantly, which indicates that AKG could promote the growth performance of Songpu mirror carp. The same results that AKG was documented to have the effect of promoting growth of Songpu mirror carp were confirmed in our previous study (15, 42). At present, the mechanism of action of AKG on the physiological effects of Songpu mirror carp is still not well understood. Nevertheless, as the precursor of glutamine synthesis and energy substances, exogenous AKG not only saves glutamine oxidized by intestinal mucosal cells in part of the diet, but also maintains the integrity of intestinal structure and function through stimulating the digestive activities (43). In addition, AKG can also affect growth axis hormone secretion through amino acid conversion of glutamate, arginine and proline, thus affecting growth performance (44). Subsequently, in order to explore the mechanism of AKG in repairing damaged intestinal structure and function, we focused on the effects of AKG on intestinal immune-related digestive enzymes and immune-related response of Songpu mirror carp in this study.

The existence of immune digestive enzymes and complement constitutes an important immune barrier for non-specific immunity (45). Our data indicated that the concentration of LZM in the intestine of common carp was decreased after the injection of *A. hydrophila*. As an alkaline enzyme which can hydrolyzes mucopolysaccharides in pathogenic bacteria, LZM represents a comprehensive nonspecific immune function in fish disease prevention. Data on our study suggested a certain degree of intestinal damage was occurred after the *A. hydrophila* challenge. Similarly, ALP has been known to be associated to fish immunity due to its hydrolytic activity and studies have shown that the content of ALP was positively correlated with the disease resistance of fish (46, 47). In our study, the injection of *A. hydrophila* caused a reduction in content of ALP, suggesting that the carp had an adverse effect on their intestinal health after infection. After AKG supplementation, the content of ALP was increased, and the unit achieved a significant effect, suggesting that AKG could cause changes in content of ALP and improve intestinal injury to a certain extent.

The results in this study were consistent with previous studies, which suggested the positive role of AKG in intestinal non-specific immunity. AKG enhanced the content of LZM and ALP may be partly attributed to the immune cell membrane structure integrity. Previous study had reported that mucosal epithelial cells could secrete antimicrobial compounds such as LZM in the intestine (48). Additionally, AKG had been proven to have an effect of promoting the growth and development of the intestine and digestive glands, thereby increasing the secretion of digestive enzymes and enhancing the content of digestive enzymes in the fish (15). Accordingly, the present study suggested the concentration of complement C4 was obviously promoted after AKG treatment. As the key components in complement system, C3 and C4 are important humoral factors in fish to resist microbial infection, which play an important role in promoting swallowing and dissolving to kill pathogens (49). Our results indicated that dietary AKG could enhance the immune function of the intestine by promoting complement C4, and it was suggested that AKG supplementation strengthens the intestinal immune function, which is partially associated with improving the innate and adaptive immune components in *A. hydrophila*-challenged carp.

In general, the intestinal inflammation response of fish is accompanied by oxidative stress and excessive production of reactive oxygen species (ROS) (50). Antioxidant enzymes, such as T-AOC, SOD and GSH-Px, have the ability to remove ROS from fish, thus maintaining the integrity of intestinal epithelial cells (51–53). The present investigation demonstrated that *A. hydrophila* challenge causes the damage to the antioxidant system. On the contrary, the rise in contents of CAT and T-AOC are paralleled by a decrease in the content of MDA in the intestine of Songpu mirror carp, indicating that AKG supplementation could reduce oxidative damage caused by *A. hydrophila* infection and increase antioxidant capacity, as well as promote the radical scavenging ability. It is worth noting that AKG supplementation had no significant effect on the activity of SOD, which was consistent with previous findings that there was a negative correlation between SOD activity and the content of lipid peroxidation products (54, 55). In addition, MDA is the end product of lipid peroxidation, which induces serious damage to the structure of biofilm, thereby affecting its normal function and even causing apoptosis (56). In this study, the contents of MDA were significantly lower after *A. hydrophila* infection, indicating that the stimulation of *A. hydrophila* caused oxidative stress injury of intestinal mucosa of Songpu mirror carp, and AKG could improve the metabolic capacity of fish, and inhibit the generation of oxygen free radicals, thereby preventing lipid peroxidation (16). This protective effect of AKG may be related to the fact that AKG acts as an intermediate in the tricarboxylic acid cycle, which can provide extra ATP to the intestinal mucosal cells (43, 57, 58). Sufficient ATP can promote electron transport in the respiratory chain and maintain the activity of the ion pump that relies on ATP to operate, thereby reducing electron leakage and intracellular sodium and calcium ion loading, and slowing the formation of free radical damage (59). Furthermore, we speculated that the effects might be partly associated with the increased gene expression of antioxidant enzymes. It was reported that antioxidant enzyme activities partly depend on their gene transcription levels in fish, and this may suggests that AKG supplementation increases the activities of antioxidant enzymes, which is partly related to the upregulation of their mRNA levels in the intestine of fish (60). However, AKG may still decompose hydrogen peroxide through other non-enzymatic oxidative decarboxylation pathways to avoid free radicals from damaging the intestinal epithelial cells, and block the subsequent release and secretion of inflammatory factors (61).

Toll-like receptors (TLRs) play an important role in preventing infection and removing bacterial pathogens. Myeloid differentiation primary response gene MyD88, as the adapter protein of most TLRs, transfers TLR signals to the intracellular pathway and results in NF-κB activation and inflammatory responses (62, 63). Studies have shown that TLR recognition of pathogens is related to the level of transcription in the tissues (64, 65). In this study, the TLR4/MyD88/NF-κB signaling pathways and its downstream activation states, including TNF-α, IL-1β, IL-6, and IL-8, were systematically detected. The results showed that the gene expression levels of TLR4 and NF-κB were significantly upregulated after the infection with *A. hydrophila*, indicating that TLR4 and NF-κB were activated. The release of several cytokines including TNF-α, IL-1β, IL-6, and IL-8 from activated immune cells occur a part of the activation of systemic host defense mechanisms. And the gene expression level of TLR4 exhibited a significant decrease with AKG supplementation, indicating that the TLR4 signaling pathway was inhibited. In addition, our data showed that the pro-inflammatory expression levels of TNF-α, IL-1β, IL-6 and IL-8 were decreased and the mRNA expression level of IL-10 was upregulated with the AKG supplementation, which confirmed that activation of the TLR4 receptor mediates downstream adapter proteins and initiates downstream pathways, leading to the release of substantial TNF-α, IL-1β, IL-6 and IL-8 (66–68). After the infection with *A. hydrophila*, AKG supplementation significantly increased the gene expression level of IL-10, which indicated that AKG could inhibit the overexpression of pro-inflammatory factors, and enable the fish to remain in a steady state under inflammatory response conditions. With experimental LPS-induced inflammation, dietary AKG inhibited NF-κB signaling pathway resulting in attenuation of inflammatory processes in piglets (17). Moreover, enhancement of oxidation performance of AKG in mice was found to be accompanied by reduced levels of pro-inflammatory factors (69). Our new finding is that AKG activates both the NF-kB signaling pathway and the TLR4 signaling pathway. It is worth noting that AKG did not promote MyD88 expression, suggesting that TLR4 performed intracellular signal transduction through a MyD88-independent pathway. Studies showed that MyD88 gene-deficient mice did not completely lose their responsiveness to the stimulation of LPS (70), which indicated that TLR4 signaling was not completely dependent on the MyD88 pathway, and could also initiate subsequent new transduction by inducing other domain adapter proteins (71, 72).

Tight junctions, composed of membrane proteins claudins and cyclin ZOs, are main connections between intestinal mucosal epithelial cells and play important roles in maintaining the mechanical integrity and normal function of the intestinal mucosal barrier (73, 74). In this study, the mRNA expression levels of claudin-1, claudin-3, claudin-7, claudin-11 and ZO-1 were upregulated after the AKG supplementation although *A. hydrophila* infection downregulated the expression of above genes, which showed that AKG supplementation could significantly increase the expression activities of tight junction structural protein genes in intestinal mucosal cells, as well as promote integrity and reduce intestinal permeability of the intestinal mucosal barrier structure. Research showed that TNF-α played a key role in the chain reaction of pro-inflammatory factors triggering tight junction damage (75). AKG is capable of promoting expression of claudins under stress, reducing the expression of TNF-α and alleviating the stress response caused by LPS stimulation to a certain extent (18), and AKG supplementation could also reshape cytoskeletal microfilaments through the MLCK signaling pathway, improve the contraction of the actin ring before tight junctions and stabilize tight junction proteins such as occludin, claudin-1, ZO-1 and their surroundings cytoskeletal protein distributions, thereby reducing intestinal permeability (76). After the pathogen invades the epithelial cells, the pathogen causes obvious epithelial cell damage by secreting cytolytic enzymes such as phospholipase, and well cell integrity could undoubtedly effectively resist the invasion of external pathogens (77). Furthermore, AKG supplementation could also inhibit the production of IL-1β, IL-6, IL-8 and other pro-inflammatory factors and reduce the damage to the tight junction structure of intestinal epithelial cells (78), which is our result confirmed. Increasing evidence had shown that intestinal immunity was closely related to damage to the tight junction structure of intestinal epithelial cells and increased intestinal mucosal permeability, and this process might be caused by the pro-inflammatory cytokines released after activation of the intestinal mucosal immune system, which directly affected the tight junctions of epithelial cells and downregulates the expression of tight junction related proteins (79).

In summary, this study investigated the effects of AKG on growth performance, intestinal antioxidant capacity and immune response after *A. hydrophila* infection in Songpu mirror carp and provided partial theoretical evidence for the underlying molecular mechanisms. Based on our data, the primary results of this study were that AKG supplementation could both promote growth performance and the activities of intestinal immune-related digestive enzymes, and AKG supplementation increased the survival rate of Songpu mirror carp infected with *A. hydrophila*. Simultaneously, AKG supplementation could also improve the intestinal antioxidant capacities of Songpu mirror carp, as well as avoid intestinal damage caused by *A. hydrophila*. Moreover, AKG supplementation promoted intestinal function and the formation of tight junction proteins, which might be related to the TLR4/MyD88/NF-κB and MLCK signaling pathways. The results in this study provide new evidence to support the use of AKG as an immunotherapeutic agent that has potentially beneficial effects on resisting infection by pathogenic bacteria.

## Data Availability Statement

The raw data supporting the conclusions of this article will be made available by the authors, without undue reservation.

## Ethics Statement

The animal study was reviewed and approved by the Committee for the Welfare and Ethics of Laboratory Animals of Heilongjiang River Fisheries Research Institute of Chinese Academy of Fishery Sciences.

## Author Contributions

DW, LW, and QX designed the study. DW carried out the experiments and wrote the manuscript. DW and ZF analysed experimental data. LW, ZF, and YZ reviewed the manuscript. JL and YZ provided technical assistance. C’aW provided technical guidance. All authors contributed to the article and approved the submitted version.

## Funding

This work was supported by the National Natural Science Foundation of China (31802305), the Central Public-interest Scientific Institution Basal Research Fund (2018HYZD0503), the China Agriculture Research System of MOF and MARA (CARS-45).

## Conflict of Interest

The authors declare that the research was conducted in the absence of any commercial or financial relationships that could be construed as a potential conflict of interest.
